# The changing clinical landscape in acupuncture for women’s health: a cross-sectional online survey in New Zealand and Australia

**DOI:** 10.1186/s12906-022-03576-3

**Published:** 2022-03-31

**Authors:** Sandro Graca, Debra Betts, Kate Roberts, Caroline A. Smith, Mike Armour

**Affiliations:** 1 Northern College of Acupuncture, York, UK; 2 NICM Health Research Institute, Western Sydney University, Penrith, Australia; 3 Translational Health Research Institute, Western Sydney University, Penrith, Australia; 4 Medical Research Institute of New Zealand (MRINZ), Wellington, New Zealand

**Keywords:** Acupuncture, Chinese medicine, Women’s health, Clinical practice, Research literacy

## Abstract

**Background:**

Acupuncture is a popular treatment for women’s health. Several trials and meta-analysis have been published in recent years on key women’s health conditions but it is unclear if this has led to any changes in clinical practice or referrals from other health professionals. The aim of this survey was to explore if, how, and why, aspects of acupuncture practice have changed since our survey in 2013.

**Method:**

An online cross-sectional survey of registered acupuncturists and Chinese Medicine practitioners in Australia and New Zealand. Questions covered the practitioner demographics and training, women’s health conditions commonly treated, modalities used, sources of information and continuing education (CE) (e.g. webinars), changes in clinical practice, and referral networks.

**Results:**

One hundred and seventy registered practitioners responded to this survey, with 93% reporting treating women’s health in the last 12 months. The majority of respondents were from Australia (60%), held a bachelors level qualification (60%), and used a traditional Chinese medicine framework (86%). Most practitioners incorporated other modalities in addition to acupuncture. Most practitioners’ referral networks were predominantly based on word of mouth for menstrual, fertility and pregnancy related conditions, with referrals from medical practitioners being much less common. More than half (57%) reported having changed their women’s health practice in the past 12 months; just over a quarter of those who changed treatment (27%) reported it was due to research findings. The most commonly used sources of information/CE used to inform treatment were webinars and conferences, while peer-reviewed journal articles were the least commonly used source.

**Conclusion:**

Acupuncture practitioners in Australia and New Zealand commonly treat women’s health conditions, but this is usually the result of women seeking them out, rather than being referred from a medical practitioner. The majority of practitioners did report changing their women’s health practice, but peer reviewed academic articles alone are not an ideal medium to convey this information since practitioners favour knowledge obtained from webinars and conferences. Academics and other clinician researchers should consider alternative means of disseminating knowledge beyond traditional academic publications and conferences, special interest groups may assist in this and also help improve research literacy.

**Supplementary Information:**

The online version contains supplementary material available at 10.1186/s12906-022-03576-3.

## Background

Acupuncture is one of the most commonly used traditional, complementary, alternative or integrative medicine therapies (TCAIM) in Australia [1] and New Zealand [2], and worldwide there are reports of increasing rates of acupuncture usage in the USA [3], UK [4], and the EU [5]. Since our team’s previous survey on acupuncture usage for women’s health in 2014 [6], a number of well-designed clinical trials and meta-analysis exploring the effectiveness of acupuncture in women’s health conditions have been published, with some studies showing positive results, while others show a lack of evidence for efficacy [7–11]. Both positive and negative findings generated significant coverage and discussion on both social and traditional media [12, 13]. However, given that patients’ decision to use acupuncture is not driven by evidence of efficacy as much as they are by recommendations from friends and family [14, 15], it is unclear if these findings, even when in the lay media, have altered rates of clinical presentation for women’s health conditions. Likewise, given the publication of these findings in highly regarded ‘mainstream’ medical journals such as the Cochrane database, this may have altered referral rates from other health professionals.

Previous research from the early to mid 2000s had shown that clinical trial results do not always change practice for acupuncture practitioners [16, 17]. Findings from our group’s recent research revealed that acupuncturists treating women’s health conditions perceived that the lack of individualization of treatments in clinical trials is a major barrier to incorporating research findings into their practice [18]. However, most of these practitioners had been in practice for over 10 years, while changes in Chinese medicine education programs [19, 20], and an interest in designing and implementing an evidence based practice curriculum [21] may have altered more recent graduates’ interest and ability to change practice to reflect the current evidence. In addition, a growing interest in post-graduate fertility training and certification [22], along with specialist organizations focusing on acupuncture research [23], its dissemination [24], and specialisation in acupuncture for fertility [15] and during pregnancy [25], may have also increased awareness and willingness to move towards a research-informed practice.

Given these developments may have impacted what conditions patients are presenting with in clinic and how the practitioner designs and implements a treatment plan, this study aimed to explore whether there have been significant changes since our last survey in 2013 on presentation rates to clinics by the public, referral patterns, what sources of information practitioners use to inform their practice, if they are implementing any changes in their practice, and what factors may be driving these changes.

## Methods

Ethical approval was obtained from Western Sydney University Human Ethics Committee (H13099—approved 5 February 2019). An online survey was developed and hosted on the Qualtrics platform.

### Participants

Invitations to participate in the survey were distributed via social media and/or email between March 2019 and May 2019 via professional organizations in New Zealand or Australia representing acupuncture and Chinese medicine including Acupuncture NZ, New Zealand Acupuncture Standards Authority (NZASA), Australian Acupuncture and Chinese Medicine Association (AACMA) and the Australian Traditional-Medicine Society (ATMS). Participants were eligible if they were currently living in New Zealand or Australia, aged over 18, either a current member of Acupuncture NZ or NZASA (if located in New Zealand) or hold current Chinese Medicine Practitioner registration with the Australian Health Practitioner Regulation Agency (AHPRA) (if located in Australia). The survey was anonymous and there was no direct contact between the researchers and respondents themselves. The survey was open from 1^st^ March 2019 till 1^st^ July 2019. The survey was closed once no new responses had been received for 7 days.

### Survey design

The content of the survey was modelled on our previous 2013 survey [6] and questions covered the practitioner demographics and training, women’s health conditions commonly treated, modalities used, sources of information/CE (e.g. webinars), changes in clinical practice, and referral networks. For this iteration of the survey, a small pilot was undertaken with four acupuncturists, and minor edits were made to wording for clarity prior to dissemination. A copy of the survey tool is available as Supplementary File 1.

### Data analysis

Data was analysed using Microsoft Excel or SPSS v27. Descriptive statistics were reported as means and standard deviations, or numbers and percentages, 95% confidence intervals were reported where appropriate. Between group comparisons were undertaken using the Chi-square test. Significance was set at *p* < 0.05. Missing data was not replaced.

For the open-ended question relating to reasons for change a qualitative descriptive approach was used to analyse and code the data [26]. A qualitative content analysis was undertaken [27]. Initial meaning units were condensed into codes by one researcher (SG). Codes with similar patterns were grouped into categories. This process and the subsequent categorization were discussed with the senior author (MA).

## Results

One hundred and seventy registered practitioners responded to this survey, with 93% reporting treating women’s health in the last 12 months. Demographically, the majority of the respondents were female (76%), aged between 35–44 years-old (36%), and practicing in Australia (60%). Most practitioners trained in Australia, held a Bachelor’s level (60%) or Master’s level qualification (21%), with ten or more years of clinical experience (65%). The traditional Chinese medicine (TCM) framework was the most commonly reported style of acupuncture used (86%) and most practitioners offered other modalities in addition to acupuncture, such as moxibustion, cupping, Chinese herbal medicine, and dietary or nutritional supplements. Table 1 outlines the demographics of respondents.

**Table 1:** Demographics and practice characteristics of respondents

	**Australia (*n* = 102)**						**New Zealand (*n* = 68)**			**Total (*n* = 170)**	
	**n**		***(%)***	**95% CI**			**n**	***(%)***	**95% CI**	**n**	***(%)***
**Gender**											
Male	20		*(19.6%)*	13.0 - 28.4			19	*(27.9%)*	18.6 - 39.6	39	*(22.9%)*
Female	82		*(80.4%)*	71.6 - 87.0			48	*(70.6%)*	58.8 - 80.1	130	*(76.5%)*
Other							1	*(1.5%)*	< 0.01 - 8.6	1	*(0.6%)*
**Age**											
18–25		1	*(1.0%)*	< 0.01 - 5.9			1	*(1.5%)*	< 0.01 - 8.6	2	*(1.2%)*
26–34		8	*(7.8%)*	3.8 - 14.9			7	*(10.3%)*	4.8 - 20.0	15	*(8.8%)*
35–44		37	*(36.3%)*	27.6 - 46.0			24	*(35.3%)*	25.0 - 47.2	61	*(35.9%)*
45–54		29	*(28.4%)*	20.6 - 37.9			18	*(26.5%)*	17.4 - 38.1	47	*(27.6%)*
55–64		22	*(21.6%)*	14.6 - 30.6			15	*(22.1%)*	13.7 - 33.4	37	*(21.8%)*
65 +		5	*(4.9%)*	1.8 - 11.3			3	*(4.4%)*	1.0 - 12.7	8	*(4.7%)*
**Practice location**											
Australia										102	*(60.0%)*
New Zealand										68	*(40.0%)*
**Years in practice**											
Less than 1 year		1	*(1.0%)*	< 0.01 - 5.9			4	*(5.9%)*	1.9 - 14.6	5	*(2.9%)*
1–5 years		16	*(15.7%)*	9.8 - 24.1			16	*(23.5%)*	14.9 - 35.0	32	*(18.8%)*
6–9 years		10	*(9.8%)*	5.2 - 17.3			11	*(16.2%)*	9.1 - 26.9	21	*(12.4%)*
10–20 years		56	*(54.9%)*	45.2 - 64.2			28	*(41.2%)*	30.3 - 53.1	84	*(49.4%)*
21 + years		19	*(18.6%)*	12.2 - 27.4			9	*(13.2%)*	6.9 - 23.5	28	*(16.5%)*
**Training location**											
New Zealand		1	*(1.0%)*	< 0.01 - 5.9			47	*(69.1%)*	57.3 - 78.9	48	*(28.2%)*
Australia		95	*(93.1%)*	86.3 - 96.9			5	*(7.4%)*	2.8 - 16.5	100	*(58.8%)*
China		23	*(22.5%)*	15.5 - 31.6			19	*(27.9%)*	18.6 - 39.6	42	*(24.7%)*
Other		14	*(13.7%)*	8.2 - 21.9			7	*(10.3%)*	4.8 - 20.0	21	*(12.4%)*
**Highest qualification**											
Diploma		11	*(10.9%)*	6.0 - 18.6			13	*(19.4%)*	11.6 - 30.6	24	*(14.3%)*
Bachelor		59	*(58.4%)*	48.7 - 67.6			41	*(61.2%)*	49.2 - 72.0	100	*(59.5%)*
Master		25	*(24.8%)*	17.3 - 34.0			11	*(16.4%)*	9.3 - 27.2	36	*(21.4%)*
Doctor		3	*(3.0%)*	0.7 - 8.7			1	*(1.5%)*	< 0.01 - 8.8	4	*(2.4%)*
PhD		3	*(3.0%)*	0.7 - 8.7			1	*(1.5%)*	< 0.01 - 8.8	4	*(2.4%)*
**Style of acupuncture**											
Five Element	21		*(20.6%)*	13.8 - 29.5			16	*(23.5%)*	14.9 - 35.0	37	*(21.8%)*
Japanese	21		*(20.6%)*	13.8 - 29.5			8	*(11.8%)*	5.8 - 21.8	29	*(17.1%)*
Traditional Chinese medicine	84		*(82.4%)*	73.7 - 88.6			63	*(92.6%)*	83.5 - 97.2	147	*(86.5%)*
Western medical acupuncture	6		*(5.9%)*	2.5 - 12.5			9	*(13.2%)*	6.9 - 23.5	15	*(8.8%)*
Other style	18		*(17.6%)*	11.4 - 26.3			8	*(11.8%)*	5.8 - 21.8	26	*(15.3%)*
**Other services**											
Microsystems acupuncture (ear / scalp, etc.)	59		*(58.4%)*	48.7 - 67.6			44	*(64.7%)*	52.8 - 75.0	103	*(60.6%)*
Chinese herbal medicine	78		*(77.2%)*	68.1 - 84.4			47	*(69.1%)*	57.3 - 78.9	125	*(73.5%)*
Western herbal medicine	14		*(13.9%)*	8.3 - 22.1			14	*(20.6%)*	12.6 - 31.8	28	*(16.5%)*
Cupping	84		*(83.2%)*	74.6 - 89.3			58	*(85.3%)*	74.8 - 92.0	142	*(83.5%)*
Tuina	40		*(39.6%)*	30.6 - 49.4			35	*(51.5%)*	39.8 - 63.0	75	*(44.1%)*
Moxibustion	89		*(88.1%)*	80.2 - 93.2			56	*(82.4%)*	71.5 - 89.8	145	*(85.3%)*
Dietary / nutritional supplements	64		*(63.4%)*	53.6 - 72.1			39	*(57.4%)*	45.5 - 68.4	103	*(60.6%)*
Tai Chi / Qi Gong	19		*(18.8%)*	12.3 - 27.6			8	*(11.8%)*	5.8 - 21.8	27	*(15.9%)*
Other	16		*(15.8%)*	9.9 - 24.3			17	*(25.0%)*	16.2 - 36.5	33	*(19.4%)*

In terms of conditions treated, 92% provided treatment for menstrual health conditions, most commonly period pain (dysmenorrhea), irregular periods, polycystic ovary syndrome (PCOS) and menstrual headaches. Once per week was the most common treatment frequency (75%), with only 10% treating twice per week or more. Seventy-six percent of respondents treated fertility related conditions, most commonly in women, for a variety of reasons including general fertility health and in conjunction with in-vitro fertilisation / assisted reproductive technology, with a minority of respondents (9%) reporting treating men for fertility issues. Eighty-five percent of respondents reported treating a wide range of pregnancy related conditions, most commonly nausea in pregnancy, back/hip or pelvic pain, breech presentation and pre-birth preparation.

### Referral networks

Most practitioners’ referral networks were predominantly based on word of mouth for menstrual, fertility and pregnancy related conditions. Despite 41% reporting working in conjunction with biomedical clinicians when treating menstrual conditions, this does not translate into a higher number of referrals to practitioners for menstrual issues (*n* = 46, 32.3%) when compared to fertility (*n* = 59, 46.1%) or pregnancy (*n* = 82, 59.4%). Nevertheless, the number of referrals from other medical practitioners for pregnancy related conditions is higher than the referrals obtained through advertising and website promotion (*n* = 76, 55.1%). The reported higher prevalence of acupuncturists working in conjunction with other CAM providers when treating menstrual conditions (58%) correlates with a higher number of referrals they receive from this group of practitioners (*n* = 77, 54%). Table 2 outlines the referral patterns by condition treated.

**Table 2:** Referral patterns by condition treated

Condition	**Menstrual** ***n* = 142**		**Fertility** ***n* = 128**		**Pregnancy** ***n* = 138**	
	n	*(%)*	n	*(%)*	n	*(%)*
**Word of mouth**	142	*(100%)*	128	*(100%)*	138	*(100%)*
**Referral from other CAM providers**	77	*(54.0%)*	82	*(64.1%)*	88	*(63.7%)*
**Referral from medical health practitioners**	46	*(32.3%)*	59	*(46.1%)*	82	*(59.4%)*
**Advertising / website promotion**	76	*(53.5%)*	69	*(53.9%)*	76	*(55.1%)*

One third of the respondents do not work with any other associate practitioner when treating menstrual conditions. Moreover, when treating fertility, most respondents (60%) work isolated from other fertility practitioners, with only 6% working onsite at a fertility clinic, while 29% of practitioners work from their own clinic, albeit closely with fertility specialists.

### Changes in practice and sources of knowledge

Over half (57%) of the participants reported having changed their women’s health practice in the past 12 months, mostly with changes to treatment approach (53%), though just over a quarter (27%) reported these changes were due to research findings. The most used sources of information the practitioners relied on to inform their treatments were webinars and conferences, while peer-reviewed journal articles were the least commonly used source. Table 3 reports on the reasons for change of treatment, broken down by practice and demographics.

**Table 3:** Reasons for the change in the treatment of women’s health

	**Diagnosis method** **or technique**		**Training and** **education (webinars, seminars, conferences)**		**Acupuncture** **style, points used,** **other treatment** **modalities added**		**Accumulated** **experience**		**Treatment** **frequency**		**Research**		**Other**	
	n	*%*	n	*%*	n	*%*	n	*%*	n	*%*	n	*%*	n	*%*
Total responses *(n* = *79)*	5	*(6.3%)*	25	*(31.6%)*	13	*(16.5%)*	17	*(21.5%)*	5	*(6.3%)*	10	*(12.7%)*	4	*(5.1%)*
Currently practicing in:														
Australia *(n* = *54)*	3	*(5.6%)*	16	*(29.6%)*	7	*(13.0%)*	14	*(25.9%)*	3	*(5.6%)*	8	*(14.8%)*	3	*(5.6%)*
New Zealand *(n* = *25)*	2	*(8.0%)*	9	*(36.0%)*	6	*(24.0%)*	3	*(12.0%)*	2	*(8.0%)*	2	*(8.0%)*	1	*(4.0%)*
Years in practice:														
Less than 1 year *(n* = *0)*	0	*(0.0%)*	0	*(0.0%)*	0	*(0.0%)*	0	*(0.0%)*	0	*(0.0%)*	0	*(0.0%)*	0	*(0.0%)*
1–5 years *(n* = *11)*	0	*(0.0%)*	2	*(18.2%)*	4	*(36.4%)*	3	*(27.3%)*	1	*(9.1%)*	1	*(9.1%)*	0	*(0.0%)*
6–9 years *(n* = *12)*	1	*(8.3%)*	4	*(33.3%)*	1	*(8.3%)*	2	*(16.7%)*	2	*(16.7%)*	1	*(8.3%)*	1	*(8.3%)*
10–20 years *(n* = *43)*	3	*(7.0%)*	13	*(30.2%)*	8	*(18.6%)*	8	*(18.6%)*	2	*(4.7%)*	6	*(14.0%)*	3	*(7.0%)*
21 + years *(n* = *13)*	1	*(7.7%)*	6	*(46.2%)*	0	*(0.0%)*	4	*(30.8%)*	0	*(0.0%)*	2	*(15.4%)*	0	*(0.0%)*
Highest qualification achieved:														
Diploma *(n* = *14)*	2	*(14.3%)*	7	*(50.0%)*	1	*(7.1%)*	3	*(21.4%)*	0	*(0.0%)*	1	*(7.1%)*	0	*(0.0%)*
Bachelor *(n* = *37)*	2	*(5.4%)*	13	*(35.1%)*	8	*(21.6%)*	7	*(18.9%)*	3	*(8.1%)*	4	*(10.8%)*	0	*(0.0%)*
Master *(n* = *24)*	1	*(4.2%)*	4	*(16.7%)*	3	*(12.5%)*	7	*(29.2%)*	2	*(8.3%)*	4	*(16.7%)*	3	*(12.5%)*
Doctor *(n* = *1)*	0	*(0.0%)*	0	*(0.0%)*	1	*(100.0%)*	0	*(0.0%)*	0	*(0.0%)*	0	*(0.0%)*	0	*(0.0%)*
PhD *(n* = *1)*	0	*(0.0%)*	0	*(0.0%)*	0	*(0.0%)*	0	*(0.0%)*	0	*(0.0%)*	0	*(0.0%)*	1	*(100.0%)*
Acupuncture training location:														
Australia *(n* = *55)*	3	*(5.5%)*	16	*(29.1%)*	7	*(12.7%)*	15	*(27.3%)*	3	*(5.5%)*	8	*(14.5%)*	3	*(5.5%)*
New Zealand *(n* = *18)*	2	*(11.1%)*	8	*(44.4%)*	4	*(22.2%)*	1	*(5.6%)*	2	*(11.1%)*	1	*(5.6%)*	0	*(0.0%)*
China *(n* = *14)*	1	*(7.1%)*	4	*(28.6%)*	4	*(28.6%)*	3	*(21.4%)*	0	*(0.0%)*	2	*(14.3%)*	0	*(0.0%)*
USA *(n* = *4)*	0	*(0.0%)*	2	*(50.0%)*	0	*(0.0%)*	0	*(0.0%)*	0	*(0.0%)*	1	*(25.0%)*	1	*(25.0%)*
UK *(n* = *3)*	0	*(0.0%)*	2	*(66.7%)*	0	*(0.0%)*	1	*(33.3%)*	0	*(0.0%)*	0	*(0.0%)*	0	*(0.0%)*
Other *(n* = *6)*	0	*(0.0%)*	2	*(33.3%)*	2	*(33.3%)*	1	*(16.7%)*	0	*(0.0%)*	1	*(16.7%)*	0	*(0.0%)*

Exploring demographic and practice factors that may have influenced the decision to change treatment, there was no significant effect of where the acupuncture training was undertaken (*p* = 0.987), number of years in practice (*p* = 0.178), the highest qualification achieved (*p* = 0.51), or if they had undertaken postgraduate study (*p* = 0.131).

## Discussion

We found that the use of peer reviewed research was the least used source of knowledge and not a major agent of change for acupuncture practitioners, which explains why very few changed their practice due to research alone, despite a substantial increase in the number of clinical trials and improvements in reporting quality of acupuncture publications over time [28–30]. Birch and colleagues [31] found a total of 1311 publications between 1991–2017 with recommendations for the use of acupuncture, especially in North America, Europe, and Australasia, made by government health institutions, national guideline, and medical specialty groups [31]. Notwithstanding, they noted that that not all these recommendations are known within the acupuncture or the mainstream medical communities [31, 32]. Consequently, Birch and colleagues proposed that there should be a greater focus on research and implementation presentations at international professional symposia [32]. A recent analysis corroborates this lack of awareness, with an appraisal of Australian guidelines in pregnancy showing significant inconsistences in the recommendation, or in some cases prohibition, of acupuncture for various conditions including nausea and vomiting, and pelvic pain [33].

Sixty-five percent of our respondents have ten or more years of experience in practice and hold bachelor’s level (60%) or master’s level (21%) qualification, in line with previous findings that 78% of Chinese medicine practitioners in Australia hold a bachelor’s degree or higher [34]. Despite a substantial minority holding post graduate degrees, this did not necessarily make them more likely to use research in their practice similar to previous findings in New Zealand naturopaths and herbalists[35], but in contrast to New Zealand massage therapists[36]. This is somewhat unexpected but it is possible that despite holding a postgraduate qualification, respondents did not necessarily feel that they had the skills to interpret research. This correlation between the ability to interpret research and incorporating this into practice has been previously observed in other complementary therapists such as homeopaths [37]. Our findings suggest that despite being experienced practitioners, those with more than 10 years’ experience are just as likely as newer graduates to change their practice. Our study found that irrespective of qualification and length of time in practice, webinars, seminars, and presentations by experts were key in shaping the acupuncture practice for Australian and New Zealand practitioners. These findings support our team’s qualitative work [18] on the importance of academic and/or clinician researchers ensuring they are not only publishing within the academy but ensuring they use reputable webinars, seminars, and conferences to reach their clinical peers, to ensure translation of research into clinical practice. These findings are similar to other complementary therapists such as New Zealand massage therapists [36] who also prioritised research that was often from secondary sources such as association journals, web articles and magazines over peer reviewed journals themselves. Likewise both Australian [38] and New Zealand [35] naturopaths tended to use seminars and newsletters in preference to peer reviewed journals. Promoting the engagement of practitioners in generating practice-based knowledge through the publication of their case studies [18] and of researchers in supporting the translation of their findings into clinical decision-making offers a solid foundation for evidence-based practice. Furthermore, research literacy and knowledge translation skills can also help practitioners to translate evidence and generate content to share on social media and inform prospective patients and the general public [38].

Previous findings from a survey of TCM practitioners in the U.K. showed that education was seen as necessary to ensure that practitioners stay updated with the medical developments in the fertility field and more than half of the respondents had taken post-qualification training in acupuncture for fertility [22]. Despite this, respondents’ opinions varied on whether the specialized training is essential, while others suggested that overspecialization could impact the holistic nature of the treatment [22]. While there are special interest groups for women’s health in Australia, New Zealand [39, 40], and the U.K. [41] thus far only North America has an organization examining and registering TCM practitioners specializing in reproductive medicine [42] and one dedicated to obstetrical acupuncture [25]. These organizations started in the USA and Canada respectively, and although most of the members are from North America, both have recently opened their examination process to practitioners worldwide, providing the applicants meet the required standards, including the continuing education units necessary to sit the exam. Engaging practitioners with organisations such as this may result in improvements in research literacy while also enabling them to contribute with new knowledge from the coalface to better reflect the complexity of acupuncture practice and inform research design accordingly [18, 43]. The two-way avenue of translation of research into practice and vice-versa includes benefits to both the profession and to the public.

Our study found that acupuncturists still treat a wide range of women’s health conditions, and that most of their referrals, especially for menstrual health, come from word of mouth, while there are a greater proportion of referrals from biomedical practitioners when treating fertility and pregnancy. However, the rate of referrals from medical practitioners remains relatively low overall, and it is unclear from our survey if these are formal inter-professional referrals or a less formal ‘it cannot hurt to try’ endorsement. The low medical referral rate may be partly due to a recently identified gap in knowledge by medical practitioners about the availability, provision, and efficacy of acupuncture, leading to less patient referrals to CAM providers [44]. Another possible contributing factor to consider is the previously identified self-perpetuating communication barriers between patients using CAM their healthcare providers [44, 45]. This is caused by patients refraining from initiating the disclosure of using CAM treatments due to their expectation that a non-judgemental conversation will be initiated by their primary care clinician, whilst the latter interprets the low levels of report by their patients as low levels of use of acupuncture and other CAM modalities [45]. These factors may contribute towards acupuncturists reporting working in relative isolation from other medical practitioners. Recent calls for greater patient-centred collaborative and integrative models [46] can potentially address previously identified barriers such as the dominance of the biomedical paradigm [47] and some clinicians’ uncertainty regarding CAM practitioners’ scope of practice [48]. Despite concerns pertaining to scientific evidence, professional regulation and safety, New Zealand healthcare professionals have a positive attitude towards CAM, with midwives recommending CAM and GPs referring patients to acupuncture [2, 49]. Patient-reported data from a maternity acupuncture service in New Zealand shows that acupuncture offers a safe and beneficial non-pharmacological treatment option for pregnant women with lumbopelvic pain [50, 51]. A recent review of RCTs on the use of acupuncture or acupressure in helping women to manage pain during labour revealed that acupuncture may increase satisfaction with pain management and reduce the use of pharmacological analgesia, while acupressure may reduce pain intensity [52]. In Australia, a program of antenatal education based on complementary therapies for labour and birth, which included acupressure [53], demonstrated a reduction of use of hospital resources and the potential to decrease birth-related healthcare costs by approximately 9% [54].

There are several important limitations to acknowledge. Firstly, like other practitioner surveys of acupuncturists in Australasia, our overall response rate was low [6, 55]. This is likely due to several factors, primarily that those who were not interested in women’s health were unlikely to fill in the survey, and secondly, due to the low priority that practitioners put on research they may not feel inclined to engage with researchers [18]. Therefore, caution must be taken in extrapolating our findings, however our key findings of lack of engagement with research are in line with previous work, both quantitively [55] and qualitatively [18]. Secondly, we did not request detailed information regarding the referral interactions with other practitioners. Referral patterns both to and from acupuncture as well as other forms of CAM are unclear. These ‘referrals’ are not commonly recorded within medical files, and tend to be verbal endorsements in support of a modality rather than a specific practitioner, suggesting it is likely that these referral interactions are different to those between medical doctors [56].

## Conclusion

Acupuncture practitioners in Australia and New Zealand commonly treat women’s health conditions, but this is usually the result of women seeking them out rather than being referred from a medical practitioner. The majority of practitioners did report changing their women’s health practice, but peer reviewed academic articles alone are not an ideal medium to convey this information since practitioners favour knowledge obtained from webinars and conferences. Academics and other clinician researchers should consider alternative means of disseminating knowledge beyond traditional academic publications and conferences, and special interest groups may assist in this and also help improve research literacy.

## Supplementary Information

**Additional file 1. **Acupuncture Treatment for Women's Health 2019.

**Additional file 2.**

## Data Availability

All data generated or analysed during this study are included in this published article and its supplementary information files.

## Abbreviations

AACMA: Australian Acupuncture and Chinese Medicine Association (AACMA)
Acupuncture NZ: Acupuncture New Zealand
ATMS: Australian Traditional-Medicine Society
CAM: Complementary and alternative medicine
GPs: General practitioners
NZASA: New Zealand Acupuncture Standards Authority (NZASA)
PCOS: Polycystic ovary syndrome
RCTs: Randomised controlled trials
TCAIM: Traditional, complementary, alternative or integrative medicine therapies
TCM: Traditional Chinese medicine
