# Supplementary material for: The changing clinical landscape in acupuncture for women’s health: a cross-sectional online survey in New Zealand and Australia
Source: BMC Complement Med Ther. 2022 Mar 31;22:94. doi: 10.1186/s12906-022-03576-3 (PMC8973511; doi:10.1186/s12906-022-03576-3)
Supplement: Supplementary file 1 — Additional file 1. Acupuncture Treatment for Women's Health 2019. [file 12906_2022_3576_MOESM1_ESM.pdf]

Acupuncture Treatment for Women's Health 2019

Start of Block: Introduction

Q1.1 Welcome and thank you for your interest to participate in a survey that examines the use of acupuncture to treat women’s health.

You are invited to take part in a survey about women’s health and how you treat those conditions. We are interested in examining the diversity of clinical practice that occurs in New Zealand and Australia among acupuncturists of different backgrounds. There is a lack of reliable knowledge describing the acupuncturists who provide treatment for women's health disorders, the types of conditions that patients commonly present with, and the characteristics of the treatment given.

These findings will inform our understanding of how acupuncture is delivered in 2019 by professional acupuncturists. This study will allow future changes in clinical practice to be monitored, and also allow comparisons to be made with other communities of acupuncturists and the design of clinically relevant research.

We would like to know more about who makes up our community of professional acupuncturists, and we are also interested in finding out a bit more about you, the practitioner. Some of the survey questions enquire about where you studied, how long you’ve been practising and what related treatments, such as herbal medicine or cupping, you use in your clinic. We are also interested in what sources of information you use to develop your treatment plans. This will give us insight into the range of backgrounds, skills and treatment modalities that are used amongst acupuncturists in New Zealand and Australia.

You are eligible to complete this survey if you meet all the following criteria:
- Aged over 18 - Are either: o A current member of Acupuncture NZ or NZASA (if you are located in New Zealand) o Hold current Chinese Medicine Practitioner registration with AHPRA (if you are located in Australia) - Have been in clinical practice at least one day per week for the past 12 months. **Most of the questions focus around your practice in the previous 12 months**. There are four sections to the survey; questions related to menstrual health, fertility, pregnancy related complaints, and some questions about you and your practice. Even if you don’t treat women’s health conditions regularly, your participation is still very important and we would appreciate it if you would be able to answer the questions about you and your practice.

This online survey will take about 15-20 minutes to complete and you will be able to save and

complete your survey at a later time if you are unable to finish it in one sitting. **We would appreciate if you could complete the survey within 2 weeks**.

Your participation is voluntary and all your answers will remain confidential, they cannot be identified as belonging to you specifically through the online survey questionnaire. Please answer all the questions as honestly and accurately as possible, as the more reliable the data is, the better picture we get of our professional community.

At the end of the survey, if you are interested, there is an option to provide your contact details for some follow up focus groups relating to a more in-depth discussion on women's health treatments. This information is stored seperately to your survey responses and the two are not linked

This survey is being conducted by Professor Caroline Smith, Dr Mike Armour and Dr Debra Betts at the NICM Health Research Institute, Western Sydney University. This survey has been approved by the Western Sydney University Human Research and Ethics Committee H13099. If you have any questions or concerns before, during or after filling in this survey please contact the lead researcher Professor Caroline Smith (caroline.smith@westernsydney.edu.au)

By completing and submitting this survey you are signifying consent for your responses to be included in the analysis. Please click the arrow button to continue.

End of Block: Introduction

Start of Block: Screening

Q2.1 In the last 12 months if you have treated any women with menstrual health conditions (such as menopause, endometriosis or painful periods); or women with fertility related conditions (such as an adjunct to IVF treatment) or women with pregnancy related conditions (such as morning sickness or turning a breech baby) then answer Yes below.

If you haven't treated these conditions in the last 12 months your information is still very important to us, answer No below and you will be taken to a section where you can give us more information about you and your practice.

Have you treated any menstrual health conditions, fertility related conditions or pregnancy related conditions *in the past 12 months*?

○ Yes (1)

○ No (2)

End of Block: Screening

Start of Block: You and your practice

Q3.1 This section allows us to report on the population of acupuncturists responding to this survey. Specific information about your individual details cannot be identified from your responses to this on line survey.

Even if you only answer some or none of the questions about women's health treatments, this information is still very important to us as it allows us to describe the diversity of practice that occurs among practitioners.

Are you:

- ○ Male (1)
- ○ Female (2)
- ○ Transgender (3)
- ○ Other (4)

Q3.2 **What is your age?**

- ○ 18-25 (1)
- ○ 26-34 (2)
- ○ 35-44 (3)
- ○ 45-54 (4)
- ○ 55-64 (5)
- ○ 65+ (6)

Q3.3 **Where are you currently practicing acupuncture?**

- ○ New Zealand (1)
- ○ Australia (2)
- ○ Other, please specify: (3) ____

Q3.4 **How many years have you been practicing as an acupuncturist?**

- ○ Less than 1 year (1)
- ○ 1-5 years (2)
- ○ 6-9 years (3)
- ○ 10-20 years (4)
- ○ 21+ years (5)

Q3.5 **How would you best describe the style(s) of acupuncture that you practice? (*please select all that apply to you*)**

- ▢ Five element (1)
- ▢ Japanese (2)
- ▢ Traditional Chinese medicine (3)
- ▢ Western medical acupuncture (4)
- ▢ Other, please specify: (5)

____

Q3.6 **What other services do you provide as part of your practice? (Select all that apply)**

- [ ] Microsystems acupuncture (ear/scalp etc.) (1)
- [ ] Chinese herbal medicine (2)
- [ ] Western herbal medicine (3)
- [ ] Cupping (4)
- [ ] Tuina (5)
- [ ] Moxibustion (6)
- [ ] Dietary/nutritional supplements (7)
- [ ] Tai Chi/QiGong (8)
- [ ] Other, please specify: (9) ____

Q3.7 **Where did you undertake your acupuncture training? (*please tick all that apply)***

- ☐ New Zealand (1)
- ☐ Australia (2)
- ☐ China (3)
- ☐ Korea (4)
- ☐ America (5)
- ☐ United Kingdom (6)
- ☐ Other, please specify: (7) ____________________

Q3.8 What is the highest qualification you hold that is relevant to Chinese medicine practice?

- ○ Diploma (1)
- ○ Bachelors degree (such as a BHSc) (2)
- ○ Masters degree (either coursework or research) (3)
- ○ Doctor of Chinese Medicine or similar (such as the Doctor of Oriental Medicine degree) (4)
- ○ PhD (in a field related to acupuncture, herbal medicine or other TCM subject) (5)

Q3.9 What are the external sources of information you use when deciding on how to treat women's health conditions ? (Drag and drop these in the order you would use them. Most commonly used at the top, least commonly used at the bottom)

______ Webinar, seminars or presentations by experts in the field (such as those offered by professional bodies) (1)

______ Conferences (either academic or practitioner focussed) (2)

______ Textbooks (3)

______ Blog posts, forums or email lists (4)

______ Research articles or summaries (eg Health CMI, Google Scholar) (5)

______ Peer support (6)

______ Other (7)

Q3.10 Have you changed your treatment of women's health in any way (e.g deciding to treat or not treat a condition, treatment frequency, using specific points etc) in the past 12 months?

○ Yes (1)

○ No (2)

Q3.11

Can you tell us about what you changed and why ?

____

____

____

____

____

Q3.12 **Please indicate how your women's health patients are referred to you (*tick all that apply*):**

	Menstrual (1)	Fertility (2)	Pregnancy (3)
Word of mouth referral from previous or current patients (1)	▢	▢	▢
Referral from complementary or alternative health (CAM) practitioners (2)	▢	▢	▢
Referral from medical health practitioners (GP, nurse, specialist) (3)	▢	▢	▢
From advertising/website promotion (4)	▢	▢	▢
Unknown referral (5)	▢	▢	▢
Other, please specify: (6)	▢	▢	▢

End of Block: You and your practice

Start of Block: Menstrual health

Q4.1
Menstrual Health

This section relates to the treatment of women with menstrual health conditions (such as menopause, period pain, PMS or heavy periods) as one of their main complaints. Answer this section if you have treated any women for these kinds of conditions **in the past 12 months.**

Have you provided treatment for menstrual health in the previous year?

- ○ Yes (1)
- ○ No (2)

Q4.2

Please indicate which of the following menstrual health conditions you have treated *in the past 12 months* and provide an estimate of the number of women seen:

	Have not treated any women in the past 12 months (1)	Treated 1-10 women (2)	Treated more than 10 women (3)
Menopause (1)	○	○	○
Primary dysmenorrhea (painful periods) (2)	○	○	○
Menorrhagia (heavy periods) (3)	○	○	○
Amenhorrea (no periods) (4)	○	○	○
Pre-menstrual syndrome (PMS) (5)	○	○	○
Irregular periods (6)	○	○	○
Polycystic ovarian syndrome (PCOS) (7)	○	○	○
Menstrual headache (8)	○	○	○
Endometriosis or secondary dysmenorrhea (9)	○	○	○
Other (please specify): (10)	○	○	○

Q4.3 **When you have treated these menstrual health conditions *in the past 12 months*, which modalities have you used? (*choose all that apply*)**

	Acupuncture (1)	Moxa (2)	Electroacupuncture (3)	Chinese herbal medicine (4)	Cupping or tuina (5)	Microsystems acupuncture (ie, ear) (6)	Tai Chi/Qi Gong (7)	Lif b ac t
Menopause (1)								
Primary dysmenorrhea (painful periods) (2)								
Amenhorrea (no periods) (3)								
Pre-menstrual syndrome (PMS) (4)								
Menorrhagia (heavy periods) (5)								
Irregular periods (6)								
Polycystic ovarian syndrome (PCOS) (7)								
Menstrual headache (8)								
Endometriosis or secondary dysmenorrhea (9)								
Other, please specify (10)								

Q4.4 **When treating menstrual health conditions, do you commonly work in conjunction with other health practitioners? (*tick all that apply*):**

- [ ] Biomedical practitioners (GP or Specialist such as gynaecologist) (1)
- [ ] Other alternative health/CM practitioners eg Naturopath (2)
- [ ] No associated practitioners (3)
- [ ] Other, please specify (4) ____

Q4.5 What is your usual treatment frequency for menstrual conditions such as period pain ?

- ○ Less than once per week (1)
- ○ Once we week (2)
- ○ Twice per week (3)
- ○ More than twice per week (4)

End of Block: Menstrual health

Start of Block: Fertility

Q5.1 Acupuncture practice for the treatment of fertility related health (such as an adjunct to IVF treatment, or for increasing natural fertility.)

Despite the focus of this survey on women's health, we recognise that in treating fertility we are often treating couples and that fertility related conditions affect both women and men. Therefore we are including some questions on men's fertility in this section. Please answer this section if you have treated any men or women for these kinds of conditions i***n the past 12 months.***

- ○ Yes (1)
- ○ No (2)

Q5.2 **Within the past 12 months, please indicate the initial reason women or men sought treatment from you to promote their fertility, and provide an estimate of the number of individuals seen:**

	Have not treated any individuals in the past 12 months (1)	Treated 1 - 10 individuals (2)	Treated more than 10 individuals (3)
Women seeking general fertility health without a specific western medical diagnosis relating to their fertility (1)	○	○	○
Women seeking treatment for specific fertility related medical rationale (such as improving their egg quality, uterine lining or follicle stimulation) (2)	○	○	○
Women seeking treatment primarily to deal with stress and promote relaxation (3)	○	○	○
Women seeking treatment only pre and post embryo transfer (4)	○	○	○
Women seeking treatment following assisted reproductive failure when no further medical treatment was recommended (5)	○	○	○
Men seeking treatment for fertility (6)	○	○	○
Other, please specify: (7)	○	○	○

Q5.3 When you have treated these fertility related conditions in the past year, which modalities have you used? (*choose all that apply*)

	Acupuncture (1)	Moxa (2)	Electroacupuncture (3)	Chinese herbal medicine (4)	Cupping or Tuina (5)	Microsystems acupuncture (ie, ear)Tai Chi/Qi Gong (6)	Tai Chi/Qi Gong (7)	Lifes a bas acup the
Women not currently undertaking assisted reproductive medical treatment (1)	☐	☐	☐	☐	☐	☐	☐	☐
Women currently undertaking assisted reproductive medical treatment (2)	☐	☐	☐	☐	☐	☐	☐	☐
Men not currently undertaking assisted reproductive medical treatment (3)	☐	☐	☐	☐	☐	☐	☐	☐
Men currently undertaking assisted reproductive medical treatment (4)	☐	☐	☐	☐	☐	☐	☐	☐

Q5.4 **In your practice, when treating fertility related conditions, do you usually work: (*tick all that apply*)**

- ☐ Onsite within a fertility unit (1)
- ☐ From your own clinic but closely with fertility specialists/units (2)
- ☐ With other complementary and alternative medicine (CAM) fertility practitioners (3)
- ☐ With no associated fertility practitioners (4)
- ☐ Other, please specify (5) ____

Q5.5 **What are the costs of treatment for women receiving acupuncture treatment in relation to fertility?**

____

Q5.6 **What costs are charged for a pre and post embryo transfer?**

____

Q5.7 **Have you made any changes to your clinical practice in the treatment of fertility over the last 5 years?**

- ○ No (1)
- ○ Yes (If yes, what changes were made?) (2) ____

End of Block: Fertility

Start of Block: Pregnancy

Q6.1 Acupuncture treatment for pregnancy related conditions

This section relates to your use of acupuncture to treat women during pregnancy, labour and in the postnatal period of 6 weeks post delivery. Answer this section if you have treated any women for these kinds of conditions in the past twelve months.

Have you provided treatment for pregnant women in the previous year?

- ○ Yes (1)
- ○ No (2)

Q6.2 **Please indicate which of the following pregnancy related conditions you have treated in the past year and provide an estimate of the number of women seen:**

	Have not treated (1)	Treated 1-10 women (2)	Treated more than 10 women (3)
Nausea in pregnancy (1)	○	○	○
Threatened miscarriage (2)	○	○	○
Back/pelvic girdle/hip pain (3)	○	○	○
Depression (4)	○	○	○
Pregnancy related headaches/migraines (5)	○	○	○
Anaemia (6)	○	○	○
Varicose veins/haemorrhoids/vulval varicosities (7)	○	○	○
Elevated blood pressure (8)	○	○	○
Itching in pregnancy (9)	○	○	○
Posterior presentation (10)	○	○	○
Breech presentation (11)	○	○	○
Pre birth treatment for labour preparation (12)	○	○	○
Induction (13)	○	○	○
Attending labour to provide acupuncture for pain relief (14)	○	○	○
Postnatal mother roasting treatment (15)	○	○	○

Caesarean scar treatment (16)	○	○	○
Breast feeding related conditions (17)	○	○	○
Postnatal depression (18)	○	○	○
Post birthing related pain (19)	○	○	○
Other, please specify (20)	○	○	○

Q6.3 **When you have treated these pregnancy related conditions in the past 12 months, which modalities have you used? (*choose all that apply*)**

	Acupuncture (1)	Moxa (2)	Electroacupuncture (3)	Chinese herbal medicine (4)	Cupping or Tuina (5)	Microsystems acupuncture (ie, ear) (6)
Nausea in pregnancy (1)	▢	▢	▢	▢	▢	▢
Threatened miscarriage (2)	▢	▢	▢	▢	▢	▢
Back/pelvic girdle/hip pain (3)	▢	▢	▢	▢	▢	▢
Depression (4)	▢	▢	▢	▢	▢	▢
Pregnancy related headaches/migraines (5)	▢	▢	▢	▢	▢	▢
Anaemia (6)	▢	▢	▢	▢	▢	▢
Varicose veins/haemorrhoids/vulval varicosities (7)	▢	▢	▢	▢	▢	▢
Elevated blood pressure (8)	▢	▢	▢	▢	▢	▢
Itching in pregnancy (9)	▢	▢	▢	▢	▢	▢
Posterior presentation (10)	▢	▢	▢	▢	▢	▢
Breech presentation (11)	▢	▢	▢	▢	▢	▢

Pre birth treatment for labour preparation (12)	☐	☐	☐	☐	☐	☐
Induction (13)	☐	☐	☐	☐	☐	☐
Attending labour to provide acupuncture for pain relief (14)	☐	☐	☐	☐	☐	☐
Postnatal mother roasting treatment (15)	☐	☐	☐	☐	☐	☐
Caesarean scar treatment (16)	☐	☐	☐	☐	☐	☐
Breast feeding related conditions (17)	☐	☐	☐	☐	☐	☐
Postnatal depression (18)	☐	☐	☐	☐	☐	☐
Post birthing related pain (19)	☐	☐	☐	☐	☐	☐
Other, please specify (20)	☐	☐	☐	☐	☐	☐

Q6.4 **Which of the following statements describes your clinical practice when treating a breech presentation in an uncomplicated pregnancy (*please select all that apply*):**

- ☐ I use/demonstrate Moxibustion to BL 67 with a traditional moxa stick (1)
- ☐ I use/demonstrate Moxibustion to BL 67 with a smokeless moxa stick (2)
- ☐ I use direct moxa on BL 67 (3)
- ☐ I use acupuncture on BL 67 (4)
- ☐ I apply press needles to BL 67 that the women can wear at home (5)
- ☐ I provide written instructions and ask the woman to continue using a moxibustion stick at home (6)
- ☐ I use individualised acupuncture treatment according to presenting condition (7)
- ☐ I provide diet and lifestyle advice (8)
- ☐ I do not treat breech presentation (9)
- ☐ Other, please specify (10) ____

Q6.5 **What best describes your clinical practice regarding maternal request for acupuncture treatment to induce labour? (Select one only)**

- ○ I provide this from 36 weeks (1)
- ○ I provide this from 38 weeks (2)
- ○ I provide this from 40 weeks (3)
- ○ I only provide this when the pulses indicate a woman is ready to start labour (4)
- ○ I only provide this 1-2 weeks prior to medical induction (5)
- ○ I only provide this 5-6 days prior to medical induction (6)
- ○ I only provide this 3-4 days prior to medical induction (7)
- ○ I only provide this 1 day prior to, or the day of, medical induction (8)
- ○ I do not provide acupuncture to induce labour (9)
- ○ Other, please specify: (10) ____

Q6.6 **Please indicate how your women's health patients are referred to you (*tick all that apply*):**

	Menstrual (1)	Fertility (2)	Pregnancy (3)
Word of mouth referral from previous or current patients (1)	☐	☐	☐
Referral from complementary or alternative health (CAM) practitioners (2)	☐	☐	☐
Referral from medical health practitioners (Midwife,GP, nurse, specialist) (3)	☐	☐	☐
From advertising/website promotion (4)	☐	☐	☐
Unknown referral (5)	☐	☐	☐
Other, please specify: (6)	☐	☐	☐

End of Block: Pregnancy

Start of Block: Thank you!
